# Social Support and the Incidence of Cognitive Impairment Among Older Adults in China: Findings From the Chinese Longitudinal Healthy Longevity Survey Study

**DOI:** 10.3389/fpsyt.2020.00254

**Published:** 2020-04-07

**Authors:** Shufei Yin, Quan Yang, Jinli Xiong, Tian Li, Xinyi Zhu

**Affiliations:** ^1^Department of Psychology, Faculty of Education, Hubei University, Wuhan, China; ^2^Center on Aging Psychology, CAS Key Laboratory of Mental Health, Institute of Psychology, Chinese Academy of Sciences, Beijing, China; ^3^Beijing Key Lab of Applied Experimental Psychology, Faculty of Psychology, Beijing Normal University, Beijing, China; ^4^State Key Laboratory of Cognitive Neuroscience and Learning, Beijing Normal University, Beijing, China; ^5^Department of Psychology, University of Chinese Academy of Sciences, Beijing, China

**Keywords:** social support, older adults, cognitive impairment, Chinese Longitudinal Healthy Longevity Survey, China

## Abstract

**Objective:**

Social support shows a protective effect against cognitive impairment in older adults. However, the longitudinal relationship between the distinct sources of social support and the incidence of cognitive impairment remains unclear. This study aims to investigate the association between different sources of social support and the incidence of cognitive impairment among older adults in China.

**Method:**

We used longitudinal data (2005–2014) from the Chinese Longitudinal Healthy Longevity Survey (CLHLS, 2005–2014, mean follow-up years 5.32 ± 2.64). In total, 5897 participants (aged 81.7 ± 9.7 years, range 65–112 years, 49.0% male) were enrolled. Cognitive impairment was measured by the Mini-Mental State Examination (MMSE). Social support included support from family and friends (marital status; contacts with family and friends; children's visits; siblings' visits, sick care; money received from and money given to children) and the availability of support from social community (social service and social security). We calculated subdistribution hazard ratios (SHR) of cognitive impairment by establishing Cox regression models, adjusting for residence, gender, age, education, participation in physical exercise, activities of daily living, smoking, drinking, negative psychological well-being, baseline cognitive function, occupation, leisure activities, and diseases.

**Results:**

During a 9-year follow-up, 1047 participants developed cognitive impairment. Participants who were married had a 16.0% lower risk of developing cognitive impairment compared to the widowed older adults after controlling for all covariates, but the protective effect of being married was no longer significant (*p* = 0.067) when additional adjustment was made for all types of social support. Children's visits were significantly associated with the risk of cognitive impairment after controlling for all types of social support and covariate variables (SHR = 0.808, 95% confidence interval, 0.669–0.975, *p* = 0.026).

**Conclusion:**

Children's visits were consistently associated with a lower incidence of cognitive impairment in Chinese older adults.

## Introduction

Social support plays an important role in late life. Previous studies have shown that social support is a strong predictor of health-related quality of life, mental health, and everyday function (1, 2). Social support has been defined as “the support accessible to an individual through social ties to other individuals, groups, and the larger community” (3). It is often divided into emotional and instrumental support (2). Emotional support usually refers to the provision of caring, empathy, trust, and love (4), and instrumental support refers to the provision of tangible goods, services, or aid (4, 5).

Accumulating evidence demonstrates a protective effect of social support against cognitive decline in older adults (6, 7). MacArthur Studies of Successful Aging found that baseline social support predicted cognitive function 7.5 years later (7). A meta-analysis reported that social support associated with global cognition and memory performance in healthy older adults (6).

Although the association between social support and cognitive function is consistently observed in older adults, the longitudinal relationship between the distinct dimensions of social support and the risk of cognitive impairment remains unclear. Many studies failed to distinguish different types of social support. For example, Andrew & Rockwood used a composite “social vulnerability index” to reflect social support (8), which included emotional, instrumental, informational support from close family members, relatives, friends, and someone others. However, the protective effects of social support may differ by the types of social support. A longitudinal study reported that emotional social support showed greater protective effects on cognitive decline than instrumental support (9). As the importance of different social support sources may vary in older adults, it is meaningful to investigate the independent impact of specific sources of social support on cognitive function. For older adults, interactions with close family members (especially those who live with them) are likely to be the most influential support resources (10). Previous studies have highlighted the effect of marital status on late-life cognition. Widowhood and being single are found to be significant predictors of cognitive impairment (11–15). A cross-sectional study in China reported that family support but not support from friends was related to cognitive function (16).

In addition, the importance of different social relationships may vary in different cultural contexts. English and Carstensen (17) suggested that as social contacts of older adults decreased, the relations with their spouses and other family members comprised an important part of their social networks. Previous studies claimed that the Chinese social network structure differed from that of Western countries, as the Chinese older adults were more likely to live with their children, and their social interactions were more family-centered (18). Social support, especially emotional support from children, is one of the most important factors affecting mental health in Chinese older adults (19). Therefore, it is important to consider how various sources of social support have different impacts on cognition in Chinese contexts.

The purpose of this study is to examine the relationship between specific sources of social support and the risk of cognitive impairment in a population-based sample of Chinese older adults. We hypothesized that the protective influence of social support on the risk of cognitive impairment would differ by support sources, and support from close family members (spouses and children) would have a greater effect than other support sources.

## Methods

### Study Population

**Figure 1:**
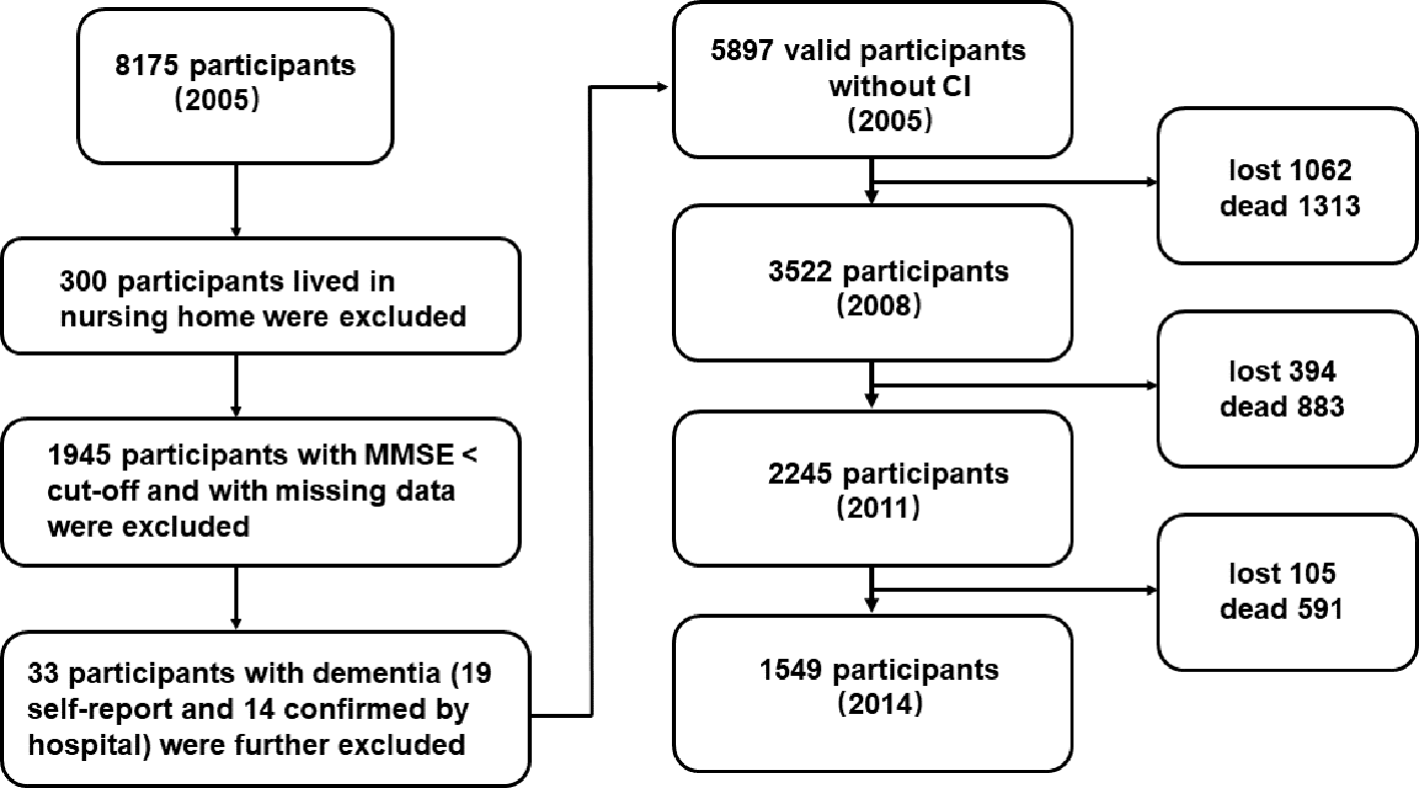
The flowchart of the study sample from 2005 to 2014. “Lost” means the data was lost in the follow-up surveys; “dead” means the participant was dead in the follow-up surveys.

Data were obtained from the Chinese Longitudinal Healthy Longevity Survey (CLHLS, http://opendata.pku.edu.cn/dataverse/CHADS). The CLHLS study was approved by the Research Ethics Committees of Duke University and Peking University. All participants provided written informed consent. The CLHLS was initiated in 1998 and follow-up surveys were conducted in 2000, 2002, 2005, 2008, 2011, and 2014. The details of the study design and data collection of CLHLS were fully described previously (20). Initially, the CLHLS project only included the oldest-old adults aged 80 and over in 22 provinces in mainland China. From 2002 onwards, the CLHLS included younger older adults aged 65–79. The present study sample included 2005–2014 longitudinal datasets. The baseline (2005) interview enrolled 8175 participants, and 300 participants who lived in nursing homes were excluded from the analysis. Then, we excluded the participants with cognitive impairment at baseline based on MMSE score, resulting in a sample of 5930 participants. In addition, 33 participants who claimed to have dementia and 14 participants who were diagnosed with dementia by the hospital were also excluded. A sample of 5897 participants with normal cognitive status was included in the analysis. **Figure 1** illustrates the flowchart of participants from baseline to the follow-up. The main reasons for the loss to follow-up were changes in home addresses and reluctance to participate due to transportation difficulties and unfavorable weather (21).

### Social Support

Social support included emotional and instrumental support from family and friends and the availability of support outside the family. Specifically, social support from family and friends included contacts with family members and friends, children's frequent visit, siblings' frequent visit, sick care (whether family members provided care when participants were in sick), money received (whether participants received money from children), and money given (whether participants gave money to their children). In addition, marital status (married and living together; married but separated; widowed; divorced; never married) as a mixed variable was also included in the analysis.

Contacts with close family members, relatives, friends, and others were measured through three questions: “the first three people you talk to when you need to tell something about yourself,” “the first three people you ask for help when you have problems/difficulties,” and “the first three people to whom you talk most frequently in daily life.” The score of contacts with close family members, relatives, friends, and others was rated according to the answers to the three questions. If the first person was the spouse, the item “spouse” scored 3; if the second person was the spouse, the item “spouse” scored 2; otherwise, the score was 1. Items like “children,” “daughter/son-in-law,” “friends,” “other relatives,” and so on were scored under the same rule as “spouse.” Composite scores were calculated separately for each item (ranging from 3 to 9). A higher score indicated closer contact. Children's and siblings' personal information (names, gender, age, relations, alive or not, and current residence) was collected, and participants were asked whether their children and siblings visited them frequently (yes or no) to evaluate the children's visits and siblings' visits. Children's visits and siblings' visits were then recoded into dichotomous variables (whether or not children/siblings visited the participant frequently). The money received from children was measured through three questions: “how much did you receive from your son(s) or daughter(s)-in-law last year?” “how much did you receive from your daughter(s) or son(s)-in-law last year?” and “how much did you receive from your grandchild(ren) last year?” Then, money received was recoded into a trichotomous variable (yes/no/unknown). The money given to children was measured in the same way. Sick care was assessed by the question “who took care of you when you were sick?” and answers were classified into four categories: none, spouse, children, others (friends, neighbors, or nurses). Then sick care was also recoded into a trichotomous variable (yes/no/unknown).

The availability of social support outside the family included the perceived availability of social services from community and social insurance. The availability of social service was assessed by asking whether a series of services (personal care, house call physicians, psychological consulting, daily shopping, social and recreation activities, legal aid, healthcare education, and mediation of neighborhood disputes) were available in the community. The availability of social insurance was assessed by asking participants whether he or she had a series of social insurance, including retirement wage, pension, private old-age insurance, public free medical services, the cooperative medical scheme, basic medical insurance, severe disease insurance, and life insurance. All answers were classified into three categories: yes, no, and unknown (**Table 1**). Then, the availability of social service and social insurance were respectively recoded into composite dichotomous variables (whether at least one social service/insurance was available).

**Table 1:** The measurement of the availability of social security and social service.

Measurements	N		
	Yes	No	Unknown
**Social security availability**			
Do you have retirement wage at present?	1385	4511	1
Do you have pension at present?	278	5619	0
Do you have private old age insurance at present?	49	5848	0
Can you access to public free medical services at present?	486	5411	0
Can you access to the cooperative medical scheme at present?	614	5283	0
Do you have basic medical insurance at present?	636	5261	0
Do you have severe disease insurance at present?	256	5641	0
Do you have life insurance at present?	67	5830	0
**Social service availability**			
Is personal care service available in your community?	128	5765	4
Is house call physician available in your community?	573	5320	4
Is psychological consulting service available in your community?	311	5583	3
Is daily shopping service available in your community?	254	5639	4
Is social and recreation service available in your community?	719	5174	4
Is legal aid service available in your community?	480	5412	5
Is healthcare education service available in your community?	634	5258	5
Is neighborhood dispute mediation available in your community?	1260	4633	4

### Cognitive Impairment

Cognitive impairment was measured by the Mini-Mental State Examination (MMSE) (22). The higher the score (0–30), the greater the cognitive ability of the participant. As most of the Chinese older adults had no formal education, several items of MMSE were simplified to make them more practical. The serial 7 subtraction was simplified to serial 3 subtraction, and reading and writing a sentence was replaced by verbally naming as many kinds of food as possible in one minute (23). As over half of the participants (54%) received no formal education in the present study, we used education-based MMSE cutoff points to define cognitive impairment: < 18, participants with no formal education; < 21, participants with 1–6 years of education; and <25, participants with more than 6 years of education (24, 25).

### Covariables

Several control variables were adjusted in Cox models, including gender, age, residence (rural, town, city), education (years of schooling), participation in physical exercise (yes/no), activities of daily living (ADL), smoking (yes/no), drinking (yes/no), negative well-being (3–15), baseline MMSE, occupation (labor/intellectual), leisure activities, and physical diseases (yes/no).

ADL ability was measured through the Katz Index of Activities of Daily Living scale (Cronbach's α = 0.87) (26). An index of negative well-being was used to control the potential influence of depressive symptoms, as no direct measure of depressive symptoms was included in the CLHLS questionnaire (25, 27). The index was measured by three items about neuroticism (“I often feel fearful or anxious”), loneliness (“I often feel lonely or isolated”), and perceived loss of self-worth (“The older I get, the more useless I feel”). Participants answered on a five-point Likert scale, with “1” for “does not describe me at all” and “5” for “describes me very well.” The sum score on three items was the score of negative well-being, with a higher score indicating worse psychological well-being. It is the recommended measurement of depressive symptoms in CLHLS database book (27). Participation in physical exercise was measured by one question: “Do you regularly participate in physical exercise (yes or no)?” Occupation was measured by one question: “What was your primary occupation before age 60?” Nine alternative answers were offered in the questionnaire: (1) professional or technical (personnel/doctors/teachers), (2) governmental, institutional or managerial personnel, (3) staff/service worker/industrial worker, (4) self-employer, (5) agriculture, forestry, animal husbandry, fishery, (6) housewife, (7) military personnel, (8) unemployed, (9) others. Among these answers, (1) and (2) were defined as “intellectual work”; (3), (5), (6), and (7) were defined as “labor work”; (4), (8), and (9) were defined as “others.” Physical diseases were measured by the question of whether participants have suffered any physical diseases, including hypertension, diabetes, heart disease, stroke and cerebrovascular disease, bronchitis, emphysema, asthma and pneumonia, pulmonary tuberculosis, cataracts, glaucoma, cancer, prostate tumor, gastric or duodenal ulcer, Parkinson's disease, bedsore, arthritis, and so on. The measurement of disease was then recoded as a dichotomous variable. In addition, the measurement of leisure activities included participants' engagement in housework, gardening, reading, playing cards/mahjong, raising pets/animals, watching TV/listening to the radio, and social activities. The answers were the frequencies of the eight activities: “almost every day,” “not daily, but at least once a week,” and “not weekly, but at least once a month,” “not monthly, but sometimes,” and “never.” For each activity, “never” scored 0, and “almost every day” scored 4. The total score of eight leisure activities was also calculated.

### Analysis

Cox models were established to estimate the subdistribution hazard ratio (SHR) and the 95% confidence interval of cognitive impairment was associated with social support. SPSS 23.0 for Windows (SPSS Inc., Chicago, IL, USA) was used to collate, recode and analyze the dataset. The final event was defined as cognitive impairment. The time of the incident was defined as the time from the 2005 investigation to the diagnosis of cognitive impairment.

First, all variables were separately included in regression models, adjusting for gender and age (**Table 3**). As the univariate analyses showed that contacts with spouse/children/children-in-law/friends/other relatives were not significantly associated with the risk of cognitive impairment, they were not included in further analyses.

Then, all types of social support (marital status, children's visits, siblings' visits, money given, and money received, sick care, the availability of social service and social security) entered regression models separately, controlling for all covariate variables. When children's visits and siblings' visits were examined, children alive and siblings alive were adjusted in the model respectively; when money given and money received were examined, children alive was also controlled; when sick care was examined, children alive and marital status were additionally adjusted in the model.

Finally, all types of social support entered the Cox regression simultaneously, controlling for all covariates, including gender, age, residence, education, negative well-being, ADL, drinking, smoking, exercise, MMSE baseline, disease, and leisure activities.

## Results

Out of 5897 participants at baseline, 1047 (17.8%) developed cognitive impairment (mean follow-up years 5.12 ± 2.32), 2266 (38.4%) were dead at the follow-up (mean follow-up years 4.57 ± 2.22), 1468 (24.9%) were lost to follow-up (mean follow-up years 3.83 ± 1.69), and 1116 (18.9%) maintained normal cognitive status (mean follow-up years 9.06 ± 0.32) at the end of the survey. **Table 2** presented the characteristics of the participants.

**Table 2:** Sample characteristics.

	CI rate (%)	Study population (n = 5897)	Status at follow-up			
			Not CI (n = 1116)	CI (n = 1047)	Dead (n = 2266)	Lost (n = 1468)
**Age**						
65–74	11.8%	1689 (28.6%)	675	199	375	440
75–84	20.0%	1957 (33.2%)	375	392	674	516
85–94	21.3%	1557 (26.4%)	59	331	791	376
95–112	18.0%	694 (11.8%)	7	125	426	136
Gender						
Female	20.2%	3009 (51.0%)	555	607	1056	791
Male	15.2%	2888 (49.0%)	561	440	1210	677
Education						
0 years	20.0%	3172 (53.9%)	533	633	1308	698
1–6 years	14.3%	2023 (34.2)	438	290	781	514
6+ years	17.7%	702 (11.9)	145	124	177	256
Residence						
Rural	19.2%	3301 (56.0%)	695	634	1387	585
City	15.3%	1284 (21.8%)	184	196	363	541
Town	16.5%	1312 (22.2%)	237	217	516	342
Marital status						
Widowed	20.5%	3253 (55.3%)	403	667	1394	789
Married	14.6%	2448 (41.5%)	669	358	783	638
Separated	9.0%	145 (2.4%)	33	13	69	30
Divorced	16.0%	25 (0.4%)	4	4	11	6
Never married	19.2%	26 (0.4%)	7	5	9	5
ADL						
Impaired (> 6)	18.7%	791 (13.3%)	17	146	420	199
Normal (6)	17.6%	5115 (86.7%)	1099	901	1846	1269
Physical exercise						
Yes	16.5%	2289 (38.7%)	385	377	875	652
No	18.6%	3608 (61.3%)	731	670	1391	816
Smoking						
Yes	15.1%	2204 (37.4%)	396	332	933	543
No	19.4%	3693 (62.6%)	720	715	1333	925
Drinking						
Yes	17.6%	1887 (31.9%)	323	332	798	434
No	17.8%	4010 (68.1%)	793	715	1468	1034
Negative well-being						
3–8	17.2%	4637 (78.3%)	918	796	1726	1177
9–15	19.6%	1293 (21.7%)	198	251	540	291
Children's visit						
Yes	17.4%	4997 (84.7%)	1005	867	1874	1251
No	20.0%	900 (15.3%)	111	180	392	217
Children alive						
Yes	17.7%	5649 (95.8%)	1089	1001	2157	1402
No	18.5%	248 (4.2%)	27	46	109	66
Siblings' visit						
Yes	15.6%	1992 (33.8%)	554	310	639	489
No	18.9%	3905 (66.2%)	562	737	1627	979
Siblings alive						
Yes	16.6%	3388 (57.4%)	846	563	1112	867
No	19.3%	2509 (42.6%)	270	484	1154	601
Money given						
Yes	15.8%	1574 (26.6%)	377	249	523	425
No	18.4%	4192 (71.1%)	716	773	1697	1006
Unknown	19.1%	131 (2.2%)	23	25	46	37
Money received						
Yes	18.1%	5015 (85.0%)	939	910	1979	1187
No	14.7%	726 (12.4%)	151	107	232	236
Unknown	19.2%	156 (2.6%)	26	30	55	45
Sick care						
Yes	17.4%	5577 (94.6%)	1075	970	2148	1384
No	24.8%	129 (2.2%)	26	32	45	26
Unknown	23.6%	191 (3.2%)	15	45	73	58
Social security						
Yes	15.2%	2256 (38.3%)	430	343	697	786
No	19.3%	3641 (61.7%)	686	704	1569	682
Social service						
Yes	17.0%	1911 (32.4%)	341	325	652	593
No	18.1%	3986 (67.6%)	775	722	1614	875
Disease						
Yes	17.9%	3245 (55.0%)	592	582	1238	833
No	17.5%	2652 (45.0%)	524	465	1028	635
Occupation						
Labor	18.3%	5034 (85.4%)	950	923	2000	1166
Intellectual	13.7%	652 (11.1%)	127	89	185	251
Others	16.6%	211 (3.5%)	39	35	81	56
Baseline MMSE						
18–20	24.5%	314 (5.3%)	21	77	145	71
21–24	24.3%	913 (15.5)	85	222	406	200
25–30	16.0%	4670 (79.2%)	1010	748	1715	1197

Married: married and living with the spouse; Separated: married and not living with the spouse.

CI, cognitive impairment; ADL, activities of daily living, MMSE, Mini-Mental State Examination.

When gender and age were adjusted, the univariate Cox regression showed that marital status, children's visits, sibling's visits, siblings alive, money given, and the availability of social insurance were significantly related to the risk of cognitive impairment, separately (**Table 3**). However, after adjusting for all covariate variables, only marital status and children's visits had significant impacts on the incidence of cognitive impairment (**Table 4**).

**Table 3:** The univariate Cox analysis of all variables (demographic variables and social support) oncognitive impairment.

Demographic variables			Social support		
	SHR (95% CI)	*p*		SHR (95% CI)	*p*
Age	1.084 (1.077–1.091)	**<0.001**	Marital status	Reference (widowed)	
Gender	0.786 (0.695–0.889)	**<0.001**	Married	0.790 (0.682–0.916)	0.002
Residence	Reference (rural)		Separated	0.379 (0.218–0.658)	**<0.001**
City	0.889 (0.756–1.045)	0.155	Divorced	0.756 (0.282–2.025)	0.578
Town	0.925 (0.793–1.080)	0.325	Never married	1.555 (0.641–3.771)	0.329
Education	0.989 (0.971–1.008)	0.271	Children's visit	0.766 (0.652–0.900)	**<0.001**
Negative well-being	1.077 (1.049–1.106)	**<0.001**	Children alive	0.824 (0.613–1.108)	0.201
ADL	1.135 (1.084–1.190)	**<0.001**	Siblings' visit	0.844 (0.734–0.972)	0.018
Drinking	1.244 (1.080–1.434)	0.003	Siblings alive	0.865 (0.758–0.986)	0.030
Smoking	0.974 (0.838–1.132)	0.730	C_spouse	0.984 (0.966–1.001)	0.068
Exercise	0.979 (0.861–1.112)	0.742	C_children	1.007 (0.985–1.030)	0.540
Disease	1.124 (0.995–1.270)	0.061	C_children in law	1.016 (0.987–1.045)	0.290
Occupation	Reference (labor)		C_relatives	0.998 (0.950–1.048)	0.923
Intellectual	0.862 (0.689–1.079)	0.195	C_friends	1.011 (0.977–1.044)	0.549
Others	0.920 (0.655–1.291)	0.629	Money given	Reference (no)	
Baseline MMSE	0.925 (0.907–0.943)	**<0.001**	Yes	0.820 (0.710–0.948)	0.007
Leisure activities	0.956 (0.945–0.967)	**<0.001**	Unknown	1.050 (0.705–1.564)	0.811
			Money received	Reference (no)	
			Yes	1.163 (0.950–1.424)	0.143
			Unknown	1.358 (0.906–2.038)	0.139
			Sick care	Reference (no)	
			Yes	0.718 (0.504–1.021)	0.065
			Unknown	1.259 (0.798–1.987)	0.322
			Social security	0.864 (0.756–0.987)	0.031
			Social service	1.026 (0.899–1.170)	0.706

All variables were included in model separately, after controlling for gender, age.

Married: married and living with the spouse; Separated: married and not living with the spouse; C_spouse: contacts with spouse; C_children: contacts with children; C_children in law: contacts with children-in-law; C_relatives: contacts with relatives; C_friends: contacts with friends and neighbors. Money given: whether participants gave money to their children or not in the past year; Money received: whether participants received money from their children or not in the past year. Sick care: whether family members' care is available or not when participants are in sick.

SHR, subdistribution hazard ratio; 95% CI, 95% confidence interval.

**Table 4:** The univariate Cox analysis of social support on cognitive impairment.

Social support	SHR (95% CI)	*p*
**Marital status**	**Reference (widowed)**	
Married	0.840 (0.722–0.976)	0.023
Separated	0.419 (0.241–0.728)	0.002
Divorced	0.666 (0.247–1.799)	0.423
Never married	1.403 (0.577–3.415)	0.455
Children's visit	0.798 (0.664–0.960)	0.017
Siblings' visit	0.906 (0.765–1.072)	0.250
Money give	Reference (no)	
Yes	0.895 (0.773–1.036)	0.138
Unknown	0.932 (0.593–1.465)	0.761
Money receive	Reference (no)	
Yes	1.088 (0.883–1.340)	0.430
Unknown	1.170 (0.745–1.837)	0.496
Sick care	Reference (no)	
Yes	0.776 (0.541–1.113)	0.168
Unknown	1.154 (0.719–1.852)	0.553
Social security	0.987 (0.849–1.148)	0.867
Social service	1.071 (0.936–1.226)	0.318

The variables were included in model separately, after controlling for gender, age, residence, negative well-being, ADL, drink, smoking, education, exercise, baseline MMSE, occupation, disease, and leisure activities. When children's visit and siblings' visit were examined, children alive and siblings alive was additional adjusted in the model respectively; when money given to and money received from children were examined, children alive was also controlled; when sick care was examined, children alive and marital status were additional adjusted in the model. For marital status, Married maps married and living with the spouse; Separated maps married and not living with the spouse.

When all covariates were controlled, the univariate Cox regression showed that participants who were married had a 16.0% lower risk of developing cognitive impairment compared to the widowed older adults (SHR = 0.840, 95% confidence interval 0.722–0.976, *p* = 0.023; **Table 4**). However, when all types of social support and covariates were included in the regression, the protective effect of being married was no longer significant (*p* = 0.067; **Table 5**).

**Table 5:** The multivariable Cox analysis of all variables on cognitive impairment.

	SHR (95%CI)	*p*
Gender	0.876 (0.739–1.039)	0.128
Age	1.066 (1.058–1.075)	**<0.001**
Residence	Reference (rural)	
City	0.932 (0.769–1.130)	0.475
Town	0.918 (0.782–1.078)	0.296
Education	1.023 (0.993–1.041)	0.056
Negative well–being	1.038 (1.009–1.068)	0.009
ADL	1.064 (1.010–1.121)	0.019
Drinking	1.261 (1.085–1.465)	0.002
Smoking	0.906 (0.772–1.064)	0.230
Exercise	1.028 (0.895–1.182)	0.692
Baseline MMSE	0.944 (0.924–0.965)	**<0.001**
Disease	1.107 (0.976–1.255)	.114
Occupation	Reference (labor)	
Intellectual	0.995 (0.752–1.315)	0.971
others	0.961 (0.682–1.354)	0.821
Leisure activities	0.972 (0.958–0.985)	**<0.001**
Marital status	Reference (widowed)	
Married	0.867 (0.744–1.010)	0.067
Separated	0.432 (0.248–0.752)	0.003
Divorced	0.599 (0.217–1.656)	0.323
Never married	1.023 (0.376–2.781)	0.964
Children's visit	0.808 (0.669–0.975)	0.026
Children alive	1.099 (0.747–1.619)	0.631
Siblings' visit	0.909 (0.767–1.077)	0.269
Siblings alive	0.991 (0.845–1.163)	0.912
Money given	Reference (no)	
Yes	0.895 (0.771–1.039)	0.144
Unknown	0.608 (0.264–1.403)	0.244
Money received	Reference (no)	
Yes	1.119 (0.905–1.384)	0.300
Unknown	1.592 (0.737–3.440)	0.237
Sick care	Reference (no)	
Yes	0.795 (0.550–1.148)	0.220
Unknown	1.168 (0.724–1.885)	0.525
Social security	1.003 (0.858–1.171)	0.972
Social service	1.071 (0.932–1.230)	0.332

All variables were included into model together. For marital status, Married maps married and living with the spouse; Separated maps married and not living with the spouse.

Children's visits had a stable impact on the incidence of cognitive impairment in univariate and multivariate analyses (**Tables 4**, **5**). Participants who were frequently visited by their children had a 19.2% lower risk of developing cognitive impairment compared to those who were not (SHR = 0.808, 95% confidence interval, 0.669–0.975, *p* = 0.026) even after controlling for all other types of social support and covariate variables.

## Discussion

In the present study, the association between social support and cognitive impairment was investigated in a representative population-based sample of Chinese older adults during a 9-year follow-up. We found that emotional support from children (children's visits) was consistently associated with a lower incidence of cognitive impairment in older adults.

With aging, the social contact of older adults decreases, and the relations with spouse and family members are a major part of their social networks (17). Thus, we hypothesized that family relations were one of the major factors that influenced cognitive function in older adults. Stable marital relationships and good relationships with children and relatives ensured daily care, family comfort, and social support for older adults (28). However, those who are widowed, divorced, or living alone lack spiritual and marital support, which may cause loneliness, insecurity, and negative attitude toward life (29), leaving them vulnerable to psychological and cognitive pathology (30).

The results partially supported the hypothesized association between family relations and the risk of cognitive impairment in Chinese older adults. Children's visits were constantly associated with a decreased risk of cognitive impairment in both univariate and multivariate analyses. Being married showed a protective effect against cognitive impairment in the univariate Cox regression compared with being widowed, but this protective effect failed to survive after controlling for other types of social support (*p* = 0.067). The result is consistent with some previous studies which found widowed older adults did not have a higher risk of cognitive impairment or dementia compared to their married counterparts (14, 31). The results also showed that older adults who were married but not living with their spouse had a lower risk of cognitive impairment compared to the widowed participants even when all types of social support and covariates were adjusted. As the number of participants who were married but not living with their spouse was relatively small (n = 145, 2.4% of the sample), the finding should be interpreted with great caution.

The main finding of the present study suggests the importance of emotional support from children in maintaining cognitive ability in Chinese older population. In general, the old parents in China lean on their children for financial support more or less. Numbers of studies found that both the provision and receipt of social support played an important role in cognitive function in older adults (32–35). Interestingly, the current study found that those participants who gave money to their children had the same risk of cognitive impairment as those who did not; also, there was no difference in the risk of cognitive impairment between participants who received money from their children and those who did not. Consistent with a previous study, Ellwardt et al. found that instrumental support did not buffer cognitive decline (9). The result might indicate that both the provision and receipt of instrumental support of children was not a vital factor in cognitive decline.

According to Berkman's theoretical model, social support refers to a person's perception of support availability in their social network (2), which does not emphasize the difference between specific resources of social support. However, different resources of social support probably play different roles in cognitive impairment. For example, a survey on Chinese older adults reported that emotional support from children is one of the most important factors in affecting mental health (19). Zhu, Hu, and Efird also found that compared to support from friends and important others, support from family was the most important indicator of older adults' cognitive function (16). However, previous studies in America reported opposite results. Brown et al. and Ficker et al. found that it was friends' support rather than family's support that had a greater impact on cognitive function of older adults (36, 37). Zhu et al. proposed that these contradictory results could be explained from the perspective of cultural differences (16).

In Chinese family culture, the social networks of older adults are more family-centered, which stresses the contact between older parents and other family members. In addition, traditional Chinese culture advocates filial piety, which is the reflection of the blood ties between parents and children in families. The traditional filial morality contributes to the development of personal morals and Chinese children are expected to take good care of and respect their parents when they are old. For many old Chinese parents, children are their important spiritual pillar and the contacts with children bring them a lot of happiness. The essence of filial piety is love, which implies gratefulness, respect, generosity, happiness, and selflessness. Numbers of studies have found that filial piety was closely associated with subjective happiness, depression, and life satisfaction (37–39). The result of the present study is in line with the expectations, and evidence suggests only children's visits, not “being married and living with the spouse,” can predict the cognitive decline in older adults. Social service, social security, or instrumental support from children cannot always predict older adults' cognitive decline, which confirmed the irreplaceable role of emotional support from children.

There are some limitations in the present study. Cognitive function was solely assessed by the MMSE, without clinical evaluation or other cognitive tests. The MMSE is a brief measure of global cognitive function, which might not be sensitive enough to screen the early stage of cognitive impairment or detect changes in cognitive function. Similarly, the measurement of social support was recoded according to the existing variables in the CLHLS questionnaire, and hence, there were unavoidable repetitions in the contents of these variables. For example, contacts with children overlapped with children's visits to some extent. However, the current analysis distinguished between the various resources of social support according to existing variables, which made a difference compared to previous studies. In addition, the measurements of social service and social security were used to assess the perceived availability of social service/security, which was not exactly the same as received social service/security. Perceived availability of social support and received social support are considered as related but different sub-constructs.

## Conclusion

In Chinese older adults, emotional support from children (children's visits) was consistently associated with a lower incidence of cognitive impairment after adjusting for all types of social support and covariates.

## Data Availability Statement

The CLHLS datasets are publicly available at the Peking University Open Research Data on CLHLS (http://opendata.pku.edu.cn/dataverse/CHADS). The dataset is publicly accessible to scholars for non-profit purposes. A signed data user agreement is required before data can be obtained.

## Ethics Statement

The study is a secondary analysis of the data from the CLHLS (http://opendata.pku.edu.cn/dataverse/CHADS), a collaborative project conducted by Duke University and Peking University. The CLHLS study was approved by the Research Ethics Committees of Duke University and Peking University. All participants provided written informed consent.

## Author Contributions

SY: study design, analysis of the raw data, interpretation of data, revision of the manuscript. QY: further data analysis, revision of the manuscript. JX: revision of the manuscript. TL: further data analysis, interpretation of data, draft and revision of the manuscript. XZ: developed the research question, interpretation of data, revision of the manuscript. All authors contributed to and have approved the final manuscript.

## Funding

This work was supported in part by National Natural Science Foundation of China (31600904), Humanities and Social Science Research Project of Hubei Provincial Department of Education (18Q017), Beijing Key Lab of Applied Experimental Psychology, Scientific Foundation of Institute of Psychology, Chinese Academy of Sciences (No. Y9CX191005) and Beijing Postdoctoral Research Foundation.

## Conflict of Interest

The authors declare that the research was conducted in the absence of any commercial or financial relationships that could be construed as a potential conflict of interest.
